# Experimental Analysis of Bragg Reflection Peak Splitting in Gratings Fabricated Using a Multiple Order Phase Mask

**DOI:** 10.3390/s19020433

**Published:** 2019-01-21

**Authors:** Gabriela Statkiewicz-Barabach, Karol Tarnowski, Dominik Kowal, Pawel Mergo

**Affiliations:** 1 Department of Optics and Photonics, Faculty of Fundamental Problems of Technology, Wroclaw University of Science and Technology, Wybrzeze Wyspianskiego 27, 50-370 Wroclaw, Poland; karol.tarnowski@pwr.edu.pl (K.T.); dominik.kowal@pwr.edu.pl (D.K.); 2 Laboratory of Optical Fiber Technology, Maria Curie-Sklodowska University, pl. M. Curie-Sklodowskiej 3, 20-031 Lublin, Poland; pawel.mergo@poczta.umcs.lublin.pl

**Keywords:** Fiber Bragg gratings, phase mask, polymer fibers

## Abstract

We performed an experimental analysis of the effect of phase mask alignment on the Bragg grating reflection spectra around the wavelength of *λ_B_* = 1560 nm fabricated in polymer optical fiber by using a multiple order phase mask. We monitored the evolution of the reflection spectra for different values of the angle ϕ by describing the tilt between the phase mask and the fiber. We observed that the peak at *λ_B_* is split into five separate peaks for the nonzero tilt and that separation of the peaks increases linearly with ϕ. Through comparison with theoretical data we were able to identify the five peaks as products of different grating periodicities, which are associated with the interference of different pairs of diffraction orders on the phase mask.

## 1. Introduction

The fabrication of fiber Bragg gratings (FBGs) in silica fibers dates back to 1978 [1], and the first successful FBG inscription in polymer optical fiber (POF) was reported in 1999 [2]. The methods for Bragg grating inscription demonstrated over the years include a phase mask technique [3,4,5,6,7,8], interferometric method [2] and point-by-point direct writing using femtosecond laser [9]. Among the others, the phase mask technique became most widely used as it allows for making gratings in a relatively simple inscription setup with modest mechanical stability requirements. However, because of the complex multi beam interference of the diffraction orders behind the phase mask, some aspects of grating formation are not completely understood yet. In particular, the role of fiber misalignment in respect to the phase mask on the reflection spectrum of the FBG is still under investigation. FBGs are often used for sensing applications where any distortion of the reflection peaks might be disadvantageous.

For FBG fabrication, specially designed phase masks are often used in order to suppress the 0^th^ diffraction order. In the case when the phase mask only supports the ±1^st^ diffraction orders, a simple interference pattern is formed by the two diffracted beams with a period equal to *Λ*/2, where *Λ* is the period of the phase mask. However, any residual power in the zero diffraction order has a significant influence on the shape of the interference pattern. Behind the phase mask, the 0^th^ order diffraction beam interferes with the ±1^st^ order beams and produces intensity modulation which in the direction parallel to the phase mask exhibits *Λ* periodicity as well as *Λ*/2 [10,11]. First observed by Talbot in 1836 [12], this effect was later analyzed by Rayleigh who calculated the period of the interference pattern in the direction perpendicular to the phase mask, named the Talbot length *Z_T_*. During the Bragg grating inscription, the intensity pattern is reproduced in the core of irradiated fiber as refractive index modulation. The existence of the Talbot-like structure of index modulation was experimentally confirmed in silica [13,14,15,16] and polymer fibers [4,6,11] irradiated through the phase mask. Besides the 0^th^ diffraction order, the Bragg grating inscription process is also affected by diffraction orders greater than 1. As reported by Smelser et al. [17], higher order diffraction on the phase mask has a significant influence on the grating characteristics even for higher order diffraction efficiency as low as 2%. Thus, most often the refractive index modulation imprinted in the fiber core consists of components with different periodicities. Moreover, higher order reflections from a single periodic component are also possible. As a result, the Bragg gratings fabricated by the phase mask method exhibit multiple peaks in their reflection spectra. It is generally assumed that the primary reflection peak in the spectrum of an FBG is the one at the wavelength *λ_B_ = n_eff_Λ*. However, observation of FBG spectra inscribed in silica fibers showed the existence of other peaks at 2*λ_B_*, 2*λ_B_*/3 and 2*λ_B_*/5 [14,18,19,20]. In [6], fabrication of FBGs with multiple reflection peaks in POFs was also demonstrated. Authors used the phase mask with 0^th^, ±1^st^, ±2^nd^ and ±3^rd^ diffraction orders, and observed reflection peaks at *λ_B_* = 1555 nm, at *λ_B_*/2 = 782 nm and 2*λ_B_*/3 = 1040 nm. 

In 1994, Dyer et al. [10] observed the splitting of Bragg reflection peak at 2*λ_B_*, which they interpreted as the result of tilting the fiber in respect to the phase mask surface. In later work [14], the peak at 2*λ_B_*/3 was also observed to split as a result of fiber tilt. The hypothetical explanation given for this effect by Yam [20] was subsequent interference blocks of *Λ* periodicity which, as written in the fiber core, constitute a type of π-shifted grating. However, this explanation was not satisfactory for Wade et al. [21] who studied experimentally the influence of fiber misalignment in reference to the phase mask during the Bragg grating inscription. In particular, the authors observed the dependence of peak separation on the tilt angle, which could not be justified by the π-shifted grating hypothesis. The origin of Bragg peak splitting was the subject of recent work by Tarnowski and Urbanczyk [22], who claimed it was an outcome of the interference of non-symmetrical diffraction orders. According to the explanation given by the authors, tilting the fiber in respect to phase mask during the grating inscription produces a few sets of periodicities of refractive index modulation imprinted in the fiber core. There will be the set of periodicities close to *Λ*, and the other set of periodicities around *Λ*/2. Depending on the number of diffraction orders, creation of periodicities close to *Λ*/3 and *Λ*/4 is also possible. Theoretical analysis pointed to linear dependence of peak separation in respect to the tilt angle for small angles, which was in good agreement with the experimental data shown in [21]. In addition, the authors predicted some effects associated with FBGs inscription in tilted fibers, which have not been observed experimentally yet, such as the formation of weak side-peaks around the Bragg peaks at *λ_B_* and 2*λ_B_*. 

In this work, we used a phase mask with the period *Λ* = 1052 nm and diffraction orders 0^th^, ±1^st^, ±2^nd^ and ±3^rd^ to fabricate gratings in a step-index PMMA (polymethyl methacrylate) fiber with a core made of PMMA/PS (polymethyl methacrylate/polystyrene) copolymer. We present a detailed experimental study of Bragg grating reflection spectra around the wavelength of λB = 1560 nm for different tilt angles set between the fiber and the phase mask. We show that with an increasing tilt angle, the reflection peak splits into five separate peaks. Among these, the side-peaks predicted in [22] were observed, which are associated with the interference between 0^th^ and ±2^nd^ diffraction orders. However, we also observed an additional pair of side-peaks which, according to our theoretical analysis, can only be second-order reflections associated with interference between 0^th^ and ±1^st^ diffraction orders. The fact that the primary Bragg peak is composed of first- as well as second-order reflections was observed earlier in [16]. The authors of this paper applied a theoretical model of reflectivity growth of FBGs presented in [23] to their experimental data and concluded there was a significant influence of second-order reflection in the peak at *λ_B_*. Our work goes farther than that as it reveals directly the composition of the central peak. We analyzed the dependence of the peak separation on the tilt angle and compared the experimental results with the theoretical predictions. We obtained a nearly perfect match for the peaks which are second-order reflections from the grating with periodicity close to *Λ* and a satisfactory match for the first-order reflection peaks from grating with periodicity close to *Λ*/2. 

## 2. Theoretical Background

**Figure 1:**
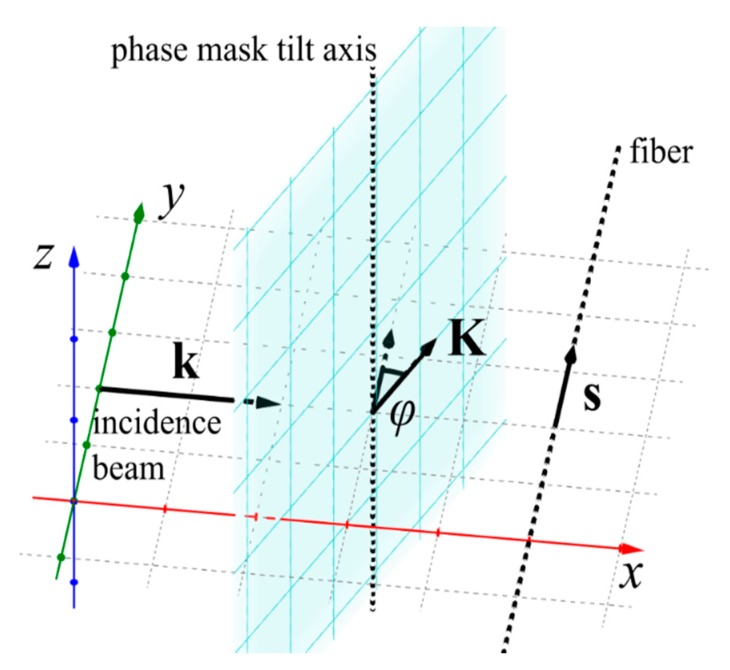
Schematic representation of grating inscription system. The blue plane represents the phase mask. ***k***, wave vector of incidence beam; ***K***, inverse vector of phase mask; ***s***, versor indicating the fiber orientation, ϕ, tilt angle.

To explain the position of observed peaks, we applied the theoretical description developed in our earlier work [22]. According to this model, we are able to calculate the periods of inscribed grating and positions of corresponding Bragg peaks. In this work, we also extend the model, by considering the higher reflection orders. For sake of clarity, we recall the model below and present its application for our experimental system. In general, the orientation of an incident beam, a phase mask and a fiber can be described with four angles as described in [22]. In our experimental system, the fiber (versor ***s*** indicates fiber direction) and the phase mask inverse vector ***K*** (|***K***|=*K*=2*π*/*Λ*, where *Λ* is phase mask period) were fixed in the incidence plane as shown in Figure 1. The wave vector of an incidence beam is denoted as ***k*** (|***k***|=k=2*π*/*λ_UV_*, where *λ_UV_* is inscribing beam wavelength). However, it was possible to tilt the phase mask by the angle ϕ. It shall be noted that phase mask rotation simultaneously changes the fiber tilt angle in respect to the phase mask and the incidence angle.

Applying the theoretical model, we calculate the periodicities present in inscribed grating and spectral positions of Bragg reflection peaks for *Λ* = 1052 nm, *λ_UV_* = 325 nm.

Using the notation introduced in [22], β is the angle between the incidence plane and the phase mask inverse vector; θ is the angle of fiber rotation around the phase mask normal with reference to the phase mask inverse vector, both equal to zero; and α is the incidence angle equal to ϕ, the angle of fiber tilt. As a result, periodicities present in the grating due to interference of *m^th^* and *q^th^* diffraction order are given by the following relation:

(1) $$\Lambda_{m,q}=\frac{\Lambda }{\left|\left(m-q\right)cos\varphi -\left(\kappa_{m}-\kappa_{q}\right)sin\varphi \right|}$$

where *m* is the component of wave vector of the *m^th^* order in the direction perpendicular to phase mask normalized to the inverse vector of phase mask (***K***):

(2) $$\kappa_{m}=\sqrt{{\left(\frac{k}{K}\right)}^{2}-{\left(\frac{k}{K}sin\varphi +m\right)}^{2}}$$

Each periodicity that is present in the grating, makes a contribution to a reflection at the wavelengths fulfilling the Bragg condition:

(3) $$\lambda_{m,q}^{\left(p\right)}=\frac{2}{p}n\left(\lambda_{m,q}^{\left(p\right)}\right)\Lambda_{m,q}$$

where *n*(*λ*) denotes the effective refractive index as the function of wavelength and $\lambda_{m,q}^{\left(p\right)}$ gives the wavelength of *p^th^* order reflection related to *Λ_m,q_* periodicity. By knowing the Bragg peak at 1560 nm is the reflection from index periodicity equal to *Λ*/2 = 526 nm for tilt angle ϕ = 0°, we are able to calculate the effective refractive index for this wavelength as *n* (1560 nm) = 1.48289. Neglecting wavelength dependency of the effective refractive index, we can then calculate the position of Bragg reflection peaks as the function of the tilt angle ϕ. 

**Figure 2:**
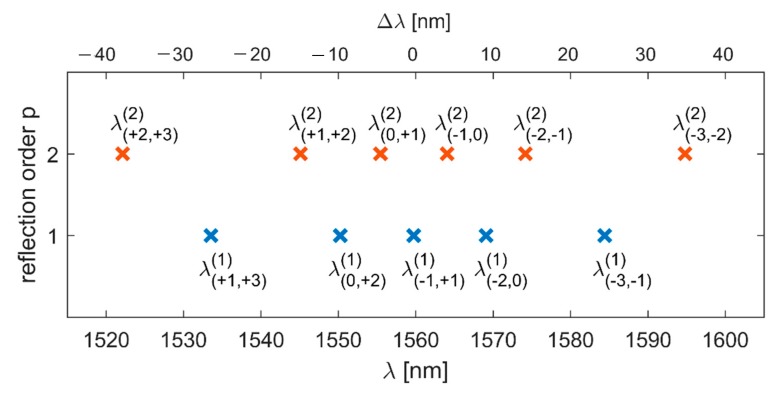
Calculated positions of Bragg reflection peaks λ_(m,q)_^(p)^, where m and q are the diffraction order number and p is the reflection order (p = 1 or 2). The auxiliary axis shows the difference between each position λ_(m,q)_^(p)^ and the position of the central peak λ_(−1,1)_^(1)^.

In our work, we focused on the splitting of the Bragg reflection peak located at 1560 nm. The applied phase mask (*Λ* = 1052 nm) under illumination with a *λ_UV_* = 325 nm beam generates seven diffracted beams (diffraction order from −3^rd^ to +3^rd^). In case of perfect alignment, there are two periodicities contributing to the Bragg reflection peak at 1560 nm: *Λ* and *Λ*/2 [16]. The observed reflection is the first-order reflection from the refractive index modulation with periodicity Λ/2 (this periodicity arises due to interference of −1^st^ and +1^st^ diffraction orders, but also other pairs which differ by 2: −3^rd^ and −1^st^; −2^nd^ and 0^th^; 0^th^ and +2^nd^; +1^st^ and +3^rd^) and the second-order reflection from the refractive index modulation with periodicity *Λ* (this periodicity arises due to interference of −1^st^ with 0^th^ and 0^th^ with +1^st^ diffraction orders, but also other pairs which differ by 1: −3^rd^ and −2^nd^; −2^nd^ and −1^st^; +1^st^ and +2^nd^; +2^nd^ and 3^rd^). According to the theoretical model, in case of nonzero tilt angle ϕ, the mentioned interfering pairs imprint different periodicities along the fiber and, as a result, the Bragg peak at 1560 nm splits. The calculated positions of Bragg reflection peaks (five arising as the first-order reflections and six arising as the second-order reflections) are presented in Figure 2 with assumed tilt angle ϕ = 1°. We also calculated the difference between the position of each peak and the central one λ_(−1,+1)_^(1)^. The comparison with experimental data will be easier with this quantity, as the positions of all peaks change slightly during inscription, due to polymer properties.

## 3. Experimental Analysis

**Figure 3:**
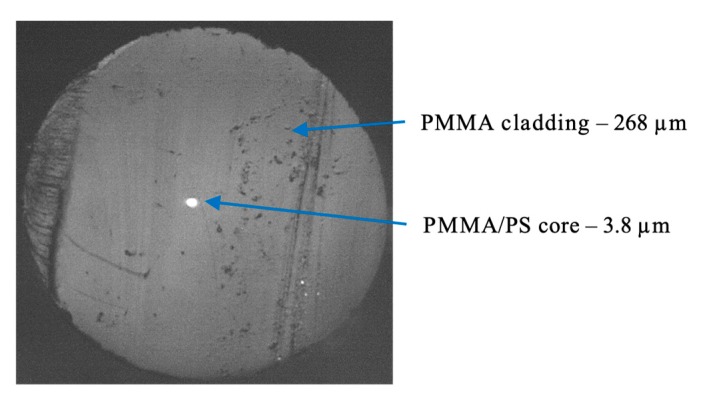
Cross section of the polymer optical fiber (POF) used for Bragg grating inscription. PMMA: polymethyl methacrylate; PS: polystyrene.

In the experiments we used a step-index POF made of PMMA in the cladding and PMMA/PS copolymer (5% *w*/*w*) in the core, which was fabricated at Maria Curie–Sklodowska University, Lublin, Poland. The outer diameter of the fiber was equal to 268 μm and the diameter of the core was 3.8 μm. The cross section of the POF is shown in Figure 3. Prior to grating inscription, the fiber was annealed at 85 °C for 10 hours which is an important technological step serving to improve the quality of the reflection spectrum of the FBG [24]. This is associated with the fact that some part of the frozen-in stress is released in the POF at elevated temperature.

**Figure 4:**
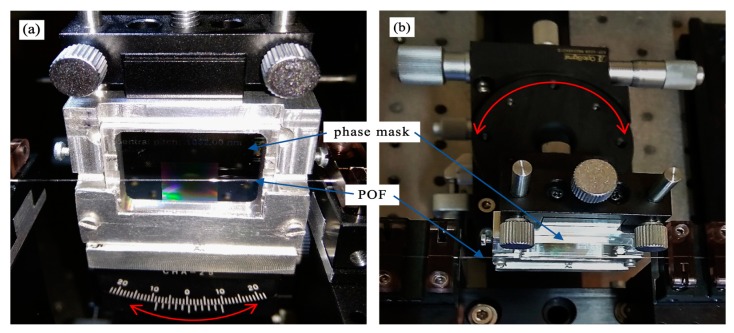
OptoSigma goniometer stage (**a**) and rotational stage (**b**) for precise adjustment of θ and ϕ angles, respectively. The directions of rotation of the phase mask relative to the POF (polymer optical fiber) are marked with red arrows.

To imprint Bragg gratings in the POF, we used a CW 30 mW He–Cd laser with emission line at 325 nm and a phase mask from Ibsen Photonics with period *Λ* = 1052 nm. The UV beam was focused on the fiber core by the use of two plano-convex lenses with focal lengths of 75 mm and 25 mm, located at 343 mm and 28 mm from the fiber surface, respectively. The resulting focus line was about 3.8 mm long. Due to high attenuation of the polymer fibers in the third telecommunication window, we used a silica fiber coupler, a supercontinuum source (SuperK Versa, NKT Photonics, Birkerød, Denmark) and an optical spectrum analyzer (AQ6370C, Yokogawa Electric Corporation, Tokyo, Japan) with resolution of 0.2 nm to monitor a reflection spectrum of the Bragg grating during the inscription process. The light was coupled onto a short piece (about 30 cm) of POF from silica fiber using index matching gel to reduce Fresnel reflection. In our current fabrication setup, the distance between the connection of POF with silica fiber and the part of the POF where grating was inscribed was about 10 cm and longer than before. The result was that the height of the peaks was low—about 10 dB—and much lower than FBG reflection spectra presented in our previous works [6,24]. Our irradiation setup was similar to the one shown in our former work [6], however some modification was introduced in order to allow precision control of the angle ϕ, which describes the rotation of the phase mask in the plane of incidence of the laser beam (Figure 4a) and the angle θ, which describes the rotation of the phase mask around phase mask normal, in the plane perpendicular to the plane of incidence of the laser beam (Figure 4b). 

**Figure 5:**
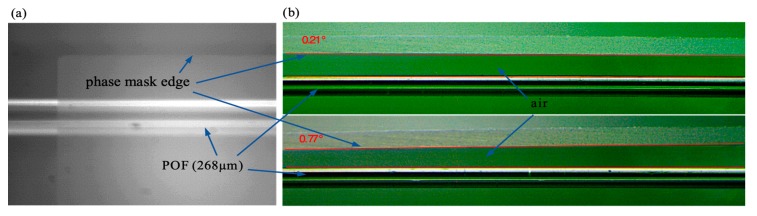
Images of the POF and the phase mask used for evaluation of the angle θ (**a**) and tilt angle ϕ (**b**).

In our experiment the θ angle was set to zero on the goniometer stage with accuracy of 0.20° and the ϕ angle was adjusted with accuracy of 0.02° by the use of a rotational table with a micrometer screw. Additionally, the fiber and the phase mask edge were observed in two planes on the camera and both angles were also measured through image analysis (Figure 5). For this purpose, one of the built-in features of DLTCamViewer software was used and the angles values were obtained with accuracy of 0.05°. In our previous setup, the POF was mounted in a specially designed base with V-groves. The phase mask was placed on the top of the base and the gap between the fiber and the phase mask was adjusted by the distance plates with precision of about 10 µm. In such a case the tilt angle could be controlled with accuracy lower than 0.05°, however the tilt angle change involved the need to unscrew the base on which the phase mask was mounted. Moreover, there was no possibility to verify the angle adjustment on the camera.

**Figure 6:**
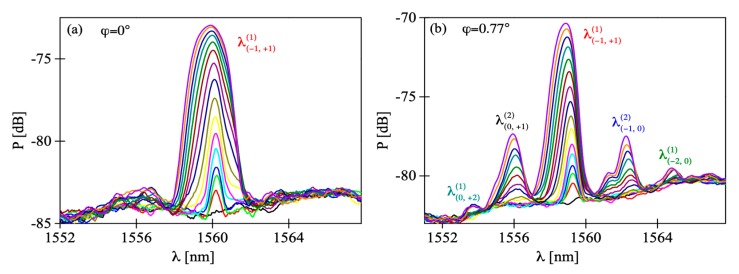
Evolution of the reflection spectra of the fiber Bragg grating (FBG) observed for different tilt angles of the POF.

**Figure 7:**
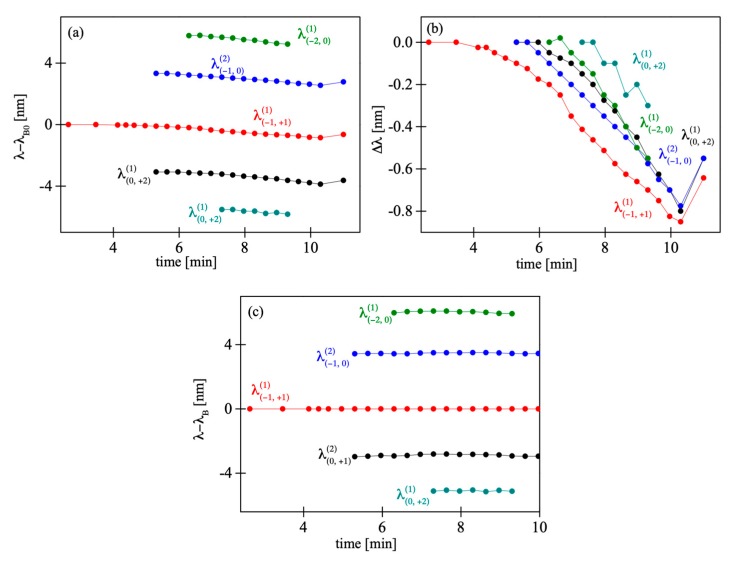
The relative position of the peaks as a function of the irradiation time for the tilt angle ϕ = 0.77°.

The fabricated FBGs exhibit a primary Bragg reflection peak at *λ_B_* = 1560 nm. In Figure 6a we show the evolution of the reflection peak of the grating during irradiation, with tilt angle ϕ set to 0°. Here, as we observed in our previous works [6,24], the peak position shifts towards shorter wavelengths as the irradiation progresses. No splitting of the reflection peak was observed, which implies the fiber was indeed parallel to the grating surface. However, when the tilt angle was increased to 0.77° it led to significant change in the grating reflection spectrum (Figure 6b). In this case, around the primary Bragg peak four smaller peaks could be observed growing above the noise level. In Figure 6a,b the peaks are denoted as $\lambda_{\left(m,q\right)}^{\left(p\right)}$, which means they are reflections of the order *p* from refractive index periodicity associated with interference of *m^th^* and *q^th^* diffraction orders. Issues concerning the peak’s identification are explained below. We studied the evolution of the Bragg peaks during the inscription process and after switching off the He-Cd laser. Precise values of distinct peaks’ positions are plotted against irradiation time in Figure 7. Figure 7a shows the relative position of the peaks with respect to the initial position of the central peak *λ_B0_*, Figure 7b shows the relative position of the peaks with respect to the initial position of each peak, and Figure 7c presents the peak separation during and after the inscription process. 

All the peaks shifted towards shorter wavelengths with increasing irradiation time, Figure 7a,b. During irradiation with a UV beam, the fiber heated up. As a consequence, a stress frozen in the fiber partially relaxed and the gratings’ periods decreased. After the He–Cd laser was turned off, all the Bragg peaks quickly moved towards longer wavelengths, which was probably caused by reorientation of molecular chains to the initial positions they had before the writing process was started [6]. This effect is not completely reversible, because the grating shrinks permanently, since the part of the frozen stress is released at increased temperatures. The dynamics is the same for all the observed peaks and, as we can see in Figure 7b, the peaks’ separation remained unchanged during the whole process (Figure 7c). 

**Figure 8:**
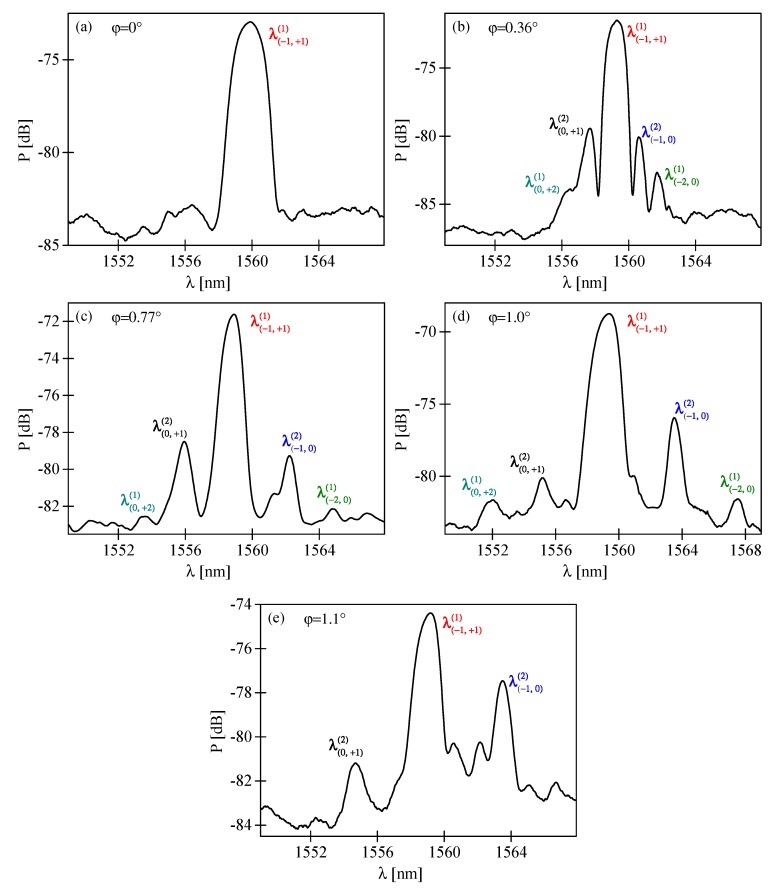
The reflection spectra near the primary Bragg wavelength *λ_B_* of the FBGs fabricated in fibers tilted in respect to the phase mask with the angle ϕ = 0° (**a**), ϕ = 0.36° (**b**), ϕ = 0.77° (**c**), ϕ = 1.0° (**d**) and ϕ = 1.1° (**e**).

The relative position of the peaks depends on phase mask orientation. We analyzed the spectra of several FBGs inscribed with different values of tilt angle ϕ. As shown in Figure 8a–e, the peaks become more distant when ϕ increases. For few tilt angles (for example Figure 8e) the outer side-peaks were not observed. The prominence of the five peaks is related to the efficiency of the interfering diffraction orders. The majority of the UV beam power (about 72%) is present in +1^st^ and −1^st^ diffraction orders (about 36% in each). Consequently, the central reflection peak dominates. The inner pair of side-peaks, associated with interference of 0^th^ and ±1^st^ diffraction orders, is significantly weaker than the central peak, because only 1.6% of laser beam power diffracts into the 0^th^ order. Moreover, the outer pair of side-peaks, associated with the 0^th^ and ±2^nd^ diffraction orders, is barely visible, due to the weakness of the 0^th^ diffraction order and low diffraction efficiency of the ±2^nd^ diffraction orders (5.5%).

**Figure 9:**
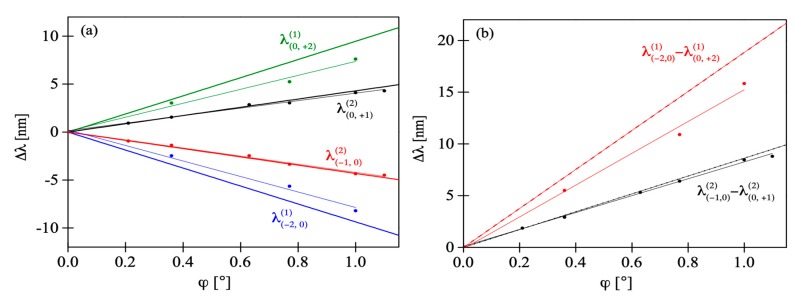
Measured and calculated shift of the side-peaks in the spectra of FBGs in respect to the position of the central peak (**a**) and the separation of the inner and outer pair of side-peaks (**b**). Experimental results are marked with circles.

According to the theoretical analysis from Section 2, when the FBG is imprinted in tilted fiber the primary Bragg peak splits into a number of separate peaks being first- or second-order reflections associated with different index periodicities. The theoretical data may be used to identify the origin of distinct peaks visible in the spectra of fabricated FBGs. As shown in Figure 2, the first peaks encountered while moving away from the central peak would be second-order reflections from index periodicities close to *Λ* associated with the interference of the 0^th^ and ± 1^st^ diffraction orders. The next pair of side-peaks would be first-order reflections from index periodicities close to *Λ*/2 associated with the interference between the 0^th^ and ±2^nd^ diffraction orders. To confirm these predictions, we analyzed the position of peaks from Figure 8. The shift of the side-peaks in respect to the position of the central peak is shown in Figure 9a and Figure 9b shows the separation of the inner and outer pairs of side-peaks, denoted $\lambda_{\left(-1,0\right)}^{\left(2\right)}-\lambda_{\left(0,+1\right)}^{\left(2\right)}$ and $\lambda_{\left(-2,0\right)}^{\left(1\right)}-\lambda_{\left(0,+2\right)}^{\left(1\right)}$. With increasing tilt, all the side-peaks move linearly apart from the central peak and apart from each other. According to [22], the position of the central peak exhibits some nonlinear shift towards shorter wavelengths with increasing ϕ but it is very weak compared to the shift of the side-peaks and was not included in Figure 9. Together with the experimental values of wavelength shift, we also show in Figure 9 the values obtained from Equation (3) and narrowed to four closest side-peaks around the central peak. The data were also fitted with straight lines and obtained slope values are given in Table 1. One can see there is a very good match between the results of the shift measured and calculated for the pair of inner side-peaks. It proves these peaks are indeed second-order reflections, as predicted above. As for the outer pair of side-peaks, the theoretical and experimental values of wavelength shift slightly diverge. Quantitative analysis shows that the slopes of straight lines *Δλ*(ϕ) fitted to the two sets of data differ by 18.5%. The reason of this difference might have been poor quality of the outer side-peaks (due to the low signal-noise ration of this measurement) which made it difficult to precisely indicate their spectral position. In fact, for some of the tilt angles the outer side-peaks were not observed, thus only three measurement points were used for comparison with theoretical data. Despite this inaccuracy, it is most probable that the origin of the outer side-peaks is first-order reflections from the index periodicities close to *Λ*/2, associated with the interference between the 0^th^ and ±2^nd^ diffraction orders. The strength of the five peaks is related to the efficiency of the interfering diffraction orders. The phase mask we used for FBG fabrication was earlier characterized in [6] where measurements of power fraction in each diffraction order were presented. The majority of power, about 35%, was accumulated in +1^st^ and −1^st^ diffraction orders and so the corresponding reflection peak is dominating. Only 1.6% of laser beam power was measured in 0^th^ diffraction order, which is why the inner pair of side-peaks, associated with interference of 0^th^ and ±1^st^ diffraction orders, is significantly weaker than the central peak. The outer pair of side-peaks, associated with the 0^th^ and ±2^nd^ diffraction orders, is barely visible, due to very weak 0^th^ diffraction order, as well as ±2^nd^ diffraction orders (5.5%). As was shown in Figure 2, the side-peaks associated with other diffraction orders would be even more distant from the central peak.

In the theoretical description, we neglect the wavelength dependence of the effective refractive index. To estimate the possible influence of this dependence we found numerically the roots of functions $f_{m,q}^{\left(p\right)}\left(\lambda \right)$ defined as follows:

(4) $$f_{m,q}^{\left(p\right)}\left(\lambda \right)=\lambda -\frac{2}{p}n\left(\lambda \right)\Lambda_{m,q}$$

We performed this calculation as the function of tilt angle ϕ for different periodicities *Λ_m,q_* and different reflection orders *p*. For this purpose, we approximated n(λ) with the refractive index of pure PMMA [25] adding a constant term to account for PS doping. This approach does not account for the waveguide contribution to effective refractive index dispersion, however, we expect that material chromatic dispersion dominates for this fiber at 1560 nm. Accounting for this dispersion in calculations shifts the Bragg peak’s position maximally by 0.27 nm for tilt angle of 2° and changes the slope of linear fit *Δλ*(ϕ) by 0.5%.

## 4. Conclusions

In this work, we presented an experimental and theoretical analysis of the spectra of FBGs fabricated by UV irradiation of POFs through the tilted phase mask. We used a phase mask with diffraction orders from –3^rd^ to +3^rd^ and a step-index PMMA fiber with copolymer of PMMA/PS in the fiber core. We modified our previously reported experimental setup in order to allow for the rotation of the phase mask in the plane of incidence of the laser beam. We studied the reflection spectrum evolution of the FBGs during the irradiation process for different phase mask alignments. We noted that the reflection peak of the fabricated FBGs was split into five distinct peaks for the nonzero tilt angles and the peak separation did not change during the grating inscription process but exhibited linear dependence on the tilt angle ϕ. By comparison with data obtained theoretically, we find that the four side-peaks observed in our experiments are first- and second-order reflections from the index periodicities close to *Λ* and *Λ*/2. The inner pair of side-peaks is associated with interference of 0^th^ and ±1^st^ diffraction orders on the phase mask while the outer pair of peaks is associated with the 0^th^ and ±2^nd^ diffraction orders. We obtained a very good match between the measured and calculated peak positions for the second-order reflection peaks and a slightly worse match for the first-order reflection peaks. In the latter case, the poor quality of the peaks is believed to be responsible for the disagreement. 

## Figures and Tables

**Table 1:** The slopes of straight lines *Δλ*(ϕ) fitted to experimental and theoretical data of the side-peaks’ shift. Fitting errors are given only for experimental data.

Experiment				Theory			
$\frac{\partial \lambda_{\left(0,+2\right)}^{\left(1\right)}}{\partial \varphi }$ [nm/°]	$\frac{\partial \lambda_{\left(-2,0\right)}^{\left(1\right)}}{\partial \varphi }$ [nm/°]	$\frac{\partial \lambda_{\left(0,+1\right)}^{\left(2\right)}}{\partial \varphi }$ [nm/°]	$\frac{\partial \lambda_{\left(-1,0\right)}^{\left(2\right)}}{\partial \varphi }$ [nm/°]	$\frac{\partial \lambda_{\left(0,+2\right)}^{\left(1\right)}}{\partial \varphi }$ [nm/°]	$\frac{\partial \lambda_{\left(-2,0\right)}^{\left(1\right)}}{\partial \varphi }$ [nm/°]	$\frac{\partial \lambda_{\left(0,+1\right)}^{\left(2\right)}}{\partial \varphi }$ [nm/°]	$\frac{\partial \lambda_{\left(-1,0\right)}^{\left(2\right)}}{\partial \varphi }$ [nm/°]
7.28 ± 0.54	−8.09 ± 0.56	3.95 ± 0.15	−4.24 ± 0.1	9.52	−9.33	4.29	−4.34
